# Effects of Shift Work in a Sample of Italian Nurses: Analysis of Rest-Activity Circadian Rhythm

**DOI:** 10.3390/ijerph18168378

**Published:** 2021-08-08

**Authors:** Letizia Galasso, Antonino Mulè, Lucia Castelli, Emiliano Cè, Vincenzo Condemi, Giuseppe Banfi, Eliana Roveda, Angela Montaruli, Fabio Esposito

**Affiliations:** 1 Department of Biomedical Sciences for Health, University of Milan, Via G. Colombo 71, 20133 Milan, Italy; antonino.mule1@unimi.it (A.M.); lucia.castelli@unimi.it (L.C.); emiliano.ce@unimi.it (E.C.); vincenzo.condemi@unimi.it (V.C.); eliana.roveda@unimi.it (E.R.); angela.montaruli@unimi.it (A.M.); fabio.esposito@unimi.it (F.E.); 2 IRCCS Istituto Ortopedico Galeazzi, Via R. Galeazzi 4, 20161 Milan, Italy; Banfi.Giuseppe@hsr.it

**Keywords:** activity levels, actigraphic monitoring, shift work, nurses, health care, occupational health

## Abstract

Shift work can lead to circadian desynchronization due to temporary misalignment between working hours and physiological and behavioral functioning, resulting in compromised health, insomnia, worsening of sleep quality, reduced ability to work during waking hours, and increased cardiovascular risk. We evaluated the effects of shift work on the rest-activity circadian rhythm (RAR) and health status of Italian orthopaedic nurses. The study population was 59 nurses: 44 worked the night shift and 15 worked the day shift. All carried out continuous 5-day actigraphic monitoring to assess RAR, including both the working and the rest period. The rhythmometric analysis showed that, during the working period, the night shift nurses had a significantly lower amplitude than the day shift nurses (*p* < 0.001), and the acrophase was significantly different between the two groups (*p* < 0.01). When we stratified the two groups by median body mass index (<25 kg/m^2^ normal weight and ≥25 kg/m^2^ overweight), during the working period, we noted a significantly lower amplitude for both the normal weight and the overweight nurses who worked the night shift (*p* < 0.01 and *p* < 0.001, normal weight and overweight respectively). The current findings suggest the need for further study of the relationship between activity levels and shift work.

## 1. Introduction

Circadian rhythmicity regulates human physiology and behavior and is involved in hormone secretion [1] and sleep [2].

Shift work, and particularly night work, can disrupt the normal sleep-wake cycle and lead to chronic desynchronization between endogenous circadian rhythms and external stimuli such as social behavior, feeding, and exercise. Under normal conditions, the endogenous sleep-wake cycle is synchronized with the day-night cycle, the timing of meals, and social routine [3,4]. Shift work involves working irregular or unusual hours different from a daytime work schedule [5]. The working hours of shift workers, because they are outside the normal daily social program, lead to circadian desynchronization due to temporary misalignment between working time and physiological and behavioral functioning, somewhat like jet lag phenomena [6]. This impacts health in various ways, including: insomnia, worsening of sleep quality, reduction in the ability to work during waking hours, reduced alertness, and increased neuromuscular fatigue, work-related stress, and cardiovascular risk [3,7,8,9,10]. The impact of shift work and its effects on worker health have been investigated in relation to cardiovascular (coronary heart disease, hypertension), metabolic (diabetes, metabolic syndrome, obesity), immune functioning [5], and mood disturbances [11]. Shift workers are also noted to be at greater risk of gastrointestinal disorders [12], workplace injuries [13], disruption in family and social life [14], and cancer [15,16].

Rest-activity circadian rhythm (RAR) refers to the level of spontaneous activity over 24 h. Since it is involved in controlling the sleep-wake cycle and numerous other physiological functions, alteration in RAR can compromise health [17,18]. RAR is described by three rhythmometric parameters (MESOR, amplitude, acrophase); RAR changes with advancing age in which amplitude is reduced and acrophase is delayed [17], similar to conditions of illness (e.g., cancer and neurological disease), in which there is a reduction in amplitude and a delay in acrophase [19,20,21], increasing the risk for cardiovascular disease [22,23].

Shift work and the associated modifications in daily routines affect the nurses’ circadian rhythm, which can lead to physical and mental health problems, and a decline in work efficiency [24,25]. Specifically in relation to the rhythmometric parameters, a recent study reported that night shift nurses in particular had less regular rest-activity cycles, greater sleep fragmentation, poorer sleep quality, displayed lower amplitude, and lower daytime activity levels [26].

Shift workers may be less likely to engage in regular physical activity, achieve complete smoking cessation, and follow a healthy diet, all modifiable risk factors for adverse health outcomes [27], including overweight and obesity [28]. Rotating night shift workers are noted to have more sedentary lifestyles and more difficulty meeting physical activity guidelines than day shift workers [29,30]. In their questionnaire-based study, Peplonska and co-workers reported that because night shift workers often have more physically demanding jobs (i.e., when assisting patients with disabilities and repositioning patients in bed), they may report more job-related physical activity and less recreational activity because of limited free time and irregular shift schedules [31], along with motivational and organizational barriers to maintaining a sufficient level of physical activity [32]. 

On the other hand, in contrast with the previous studies, Loef’s study [33] reported similar physical activity levels between nurses following different shifts, underlying that the absence of night shift does not result in less sedentary behavior and more physical activity during leisure time.

To the best of our knowledge, few studies to date have investigated the relationship between altered RAR and health in shift workers. To fill this gap, we evaluated in a sample of orthopaedic nurses the effects of irregular or unusual work hours on RAR and health status.

## 2. Materials and Methods

### 2.1. Participants

The present study involved 120 orthopaedic nurses at the Galeazzi Orthopaedic Institute (Milan, Italy). 

Inclusion criteria were:

- At least one year experience of shift work (day/afternoon or night) to guarantee an adaptation and synchronization to the shift by the nurses;
- Willingness to perform actigraphic monitoring for 5 consecutive days and to maintain a daily diary during the period.

Exclusion criteria were:

- Cardiovascular, endocrine or neuromuscular diseases (self-reported);
- Pharmacological therapy affecting sleep quality (self-reported);
- Pregnancy (self-reported).

**Figure 1:**
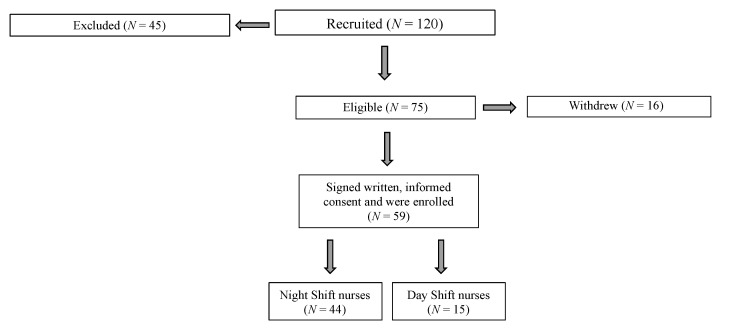
Participant recruitment. Study design, participant adherence, and dropout.

Forty-five of the 120 nurses did not meet the inclusion criteria; 16 of the remaining 75 nurses declined to participate (Figure 1). 

After receiving an explanation of the purpose of the study, 59 participants gave their written, informed consent and were enrolled in the study (November 2018 February 2019). Participants were free to withdraw from the study at any time. 

**Figure 2:**
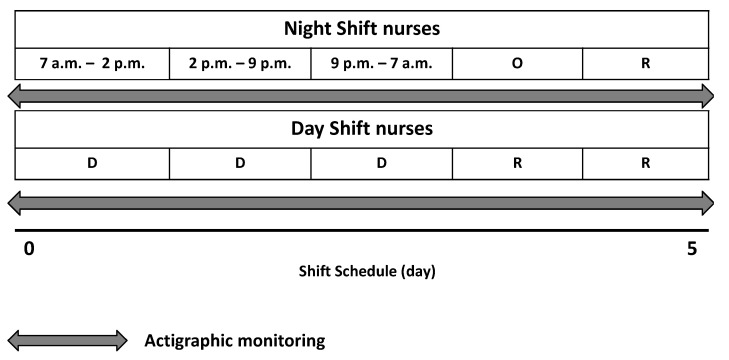
Shift schedule for night shift (NS) and day shift (DS) nurses. The grey indicators denote the duration of actigraphic monitoring. O: night-off; R: rest; D: diurnal shift.

Based on their shift schedule, the participants were divided into two groups (Figure 2): night shift nurses (NS, *n* = 44; 35 women and 9 men; mean age 41.5 ± 10.1 years; mean body mass index (BMI, weight in kg divided by the height in meters squared) 25.1 ± 3.4 kg/m^2^) and day shift nurses (DS, *n* = 15; 13 women and 2 men; mean age 40.6 ± 10.7 years; mean BMI 25.1 ± 5 kg/m^2^). The night shift group changed work shift every day over a period of 5 days, including regular work hours from 07:00 a.m. to 02:00 p.m. (day 1), from 02:00 p.m. to 09:00 p.m. (day 2), and one night shift from 09:00 p.m. to 07:00 a.m. (day 3), followed by one night off (day 4), and one day of rest (day 5). The NS group was labelled as such since the nurses work at least one night duty [34]. In addition, the categorization NS group was used to differentiate it from the DS group that worked the day shift for 3 days, from 07:00 a.m. to 02:00 p.m. or from 02:00 p.m. to 09:00 p.m., followed by two days of rest, without night duty and without work the same shift for two consecutive days. This shift cycle was maintained by both groups.

Participants responded to a brief survey investigating demographic and anthropometric characteristics, health status, and current medications. To ensure accurate actigraphic monitoring, participants were instructed in how to work the actigraphy unit and compile the daily diary. After completing one entire work shift cycle, and before starting the next one, they returned the actigraph and the diary. To avoid bias in the actigraphic analysis, we recruited nurses with at least one year of regular shift work; they were asked to maintain their regular work cycle during actigraphic monitoring and not to change shifts. 

The study was approved by the Ethical Committee of the San Raffaele Hospital (CE: 156/INT/2017), registered at ClinicalTrials.gov (registration number: NCT03453398, date 7 February 2018), and carried out in accordance with the Ethical Statements of the last Helsinki Declaration. Written, informed consent was obtained from all of the participants who were enrolled in the study. 

### 2.2. Study Design

For this study, all 59 participants were evaluated for:

- Demographic data, health status, ongoing pharmacological therapy;
- Height and body mass to calculate body mass index (BMI, kg/m^2^) [35];
- Actigraphic monitoring to record RAR. The actigraph (MotionWatch 8^®^, CamNtech, Cambridge, UK) was worn on the non-dominant wrist: the participants were instructed to remove it only when bathing, showering, washing dishes or when engaging in contact or combat sports. A daily diary was provided for entering the clock time when not wearing the actigraph and napping. Based on the data recorded in the diaries, the participants removed the actigraph once a day for about 30 min on average. The time during which the device was not worn (e.g., during personal hygiene) was deleted from the data analysis.

Actigraphic monitoring started immediately before the shift cycle began (from 07:00 a.m. to 02:00 p.m. for 5 consecutive days) and was removed before starting the next work cycle (Figure 2). During actigraphic monitoring, the participants performed their usual work and maintained their usual daily activity without following a structured protocol of physical activity.

### 2.3. Experimental Procedures

#### 2.3.1. RAR Assessment

RAR was non-invasively monitored by means of actigraphic data. The actigraph contains a piezoelectric triaxial accelerometer that records and converts axial movements over time in electrical signals. Data are collected in 30-s epochs and transferred from the Actiwatch to a personal computer using Actiwatch Analysis Software (Motion Ware 1.2.28, CamNtech, Cambridge, UK) for further analysis. Actiwatch Software was used to obtain locomotor activity data, expressed in activity counts (a.c.) and recorded every 30 s for the duration of monitoring (5 days). In order to determine the RAR, the activity data recorded by the actigraph were analyzed using the single cosinor method [36,37]. This method describes the time course of activity by means of an oscillatory, harmonic function f(t) = M + A cos (ωt + φ). The function hinges on three parameters (MESOR (M)-Midline Estimating Statistic of Rhythm; A is the amplitude; φ is the acrophase) that provide a parametric portrait of the activity rhythm for a subject. Briefly, the MESOR is the rhythm adjusted mean that approximates the arithmetical mean of the data for a given period, e.g., in the case of a single cosine approximation, the value mid-way between the highest and lowest values of the function used to approximate a rhythm. The M is equal to the arithmetic mean for equidistant data covering an integral number of cycles. The units for M are original physiologic units [38].

The amplitude is the measure of one-half the extent of the rhythmic variation in a cycle estimated by the sinusoidal or other function used to approximate the rhythm, e.g., the difference between maximum and MESOR of a best-fitting cosine. The units for amplitude are original physiologic units [39,40]. 

The acrophase indicates the time interval within which the highest values of the activity are expected. The units of the acrophase could be angular measures, degrees, radians, time units (seconds, minutes, hours, days, months, years), or physiologic episodic units. Angular measures are directly applicable to any cycle length and, hence, are proposed for general use because of greater familiarity; degrees are preferred over radians [41,42]. 

**Figure 3:**
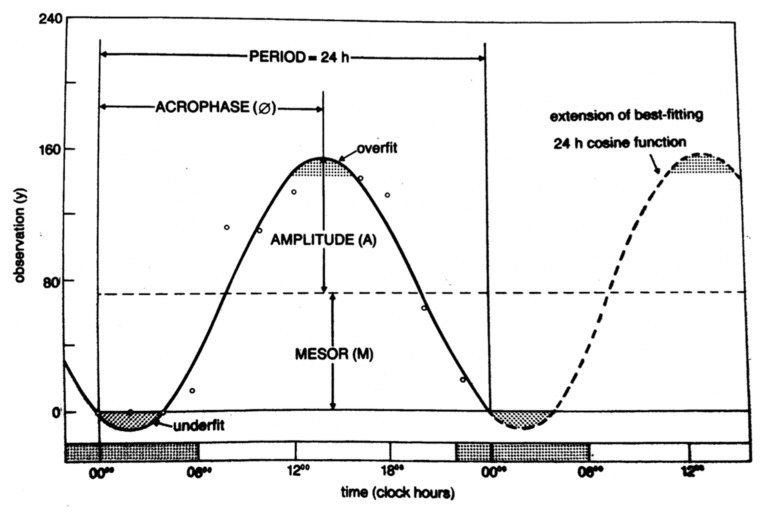
Example of a biological rhythm and its parameters: Acrophase (φ), Amplitude (A) and MESOR (M) [36].

The quantity ω is the angular frequency of oscillation and corresponds to the ratio 2π/T, where T is the period of oscillation. Confidence intervals also should be estimated for rhythm parameters (Figure 3).

#### 2.3.2. Data Processing 

For each day, the activity counts were calculated over several periods:

- Entire working cycle: all 5 days of working time;
- Working period: day 1 (morning shift 07:00 a.m.–02:00 p.m.), day 2 (afternoon shift 02:00 p.m.–09:00 p.m.), day 3 (night shift 09:00 p.m.–07:00 a.m.) for the NS group, and the 3 consecutive workdays (morning shift 07:00 a.m.–02:00 p.m. or afternoon shift 02:00 p.m.–09:00 p.m.) for the DS group;
- Rest period: day 4 (night-off) and day 5 (rest) for the NS group and the 2 consecutive rest days for the DS group.

For all of the working periods, the rhythmometric parameters were assessed based on the two-shift work schedule as a whole, NS and DS group, and then stratified by BMI.

### 2.4. Statistical Analysis 

The sample size and its statistical power were calculated in view of the main objective of the study. Calculation was performed using the analysis of variance (ANOVA) test as reference model. A number of 59 participants was expected to guarantee a statistical power > 0.80, a value of α < 0.05, and an effect size of 0.43 (G Power software, version 3.1.9.4, HHU–Düsseldorf, Düsseldorf, Germany).

Statistical analysis was carried out using R statistics software (Version 3.6.0, R Core Team. R Foundation for Statistical Computing, Vienna, Austria) [43]. Rhythmometric parameters are expressed as the mean with 95% confidence interval (CI) and the level of significance was set at α < 0.05. The Shapiro-Wilk and Kolmogorov-Smirnov tests were used to assess data normality and the Levene test was used to assess data homogeneity for all the variables analyzed. 

In order to evaluate RAR, the activity data collected with the actigraph were analyzed using the single cosinor method to identify the three rhythmometric parameters (MESOR, amplitude, acrophase). The rhythmometric parameters were then processed using the population mean cosinor. In this way, we were able to evaluate the rhythmometric characteristics of activity levels in the population and define the rhythmometric parameters of the two groups. The rhythmometric parameters of the two groups were compared using the Hotelling T^2^ test, a generalization of the multivariate field of the Student’s *t*-test. Finally, Cohen’s *d* was used to measure effect sizes. Effect sizes were interpreted according to the criteria suggested by Cohen [44] (*d* = 0.2 small; *d* = 0.5 medium; *d* = 0.8 large). Data from the demographic and anthropometric survey and actigraphic monitoring were complete. 

To determine the impact and the effect of shift work on health, because obesity can increase the risk of abnormalities in RAR and circadian disruption can interfere with the metabolic and nutritional balance of the body, we stratified the entire sample by median BMI (25 kg/m^2^) and formed two groups: a normal weight group (<25 kg/m^2^, 19 NS and 9 DS nurses) and an overweight group (≥25 kg/m^2^, 25 NS and 6 DS nurses). 

## 3. Results

The assumptions for normality and homogeneity were confirmed for all of the variables analyzed in the study.

### 3.1. Rhythmometric Analysis in Night Shift and Day Shift Nurses during the Entire Working Cycle

Table 1 reports the single cosinor method that revealed a statistically significant RAR (*p* < 0.001) for all participants. The population mean cosinor showed a significant circadian rhythm in both groups (*p* < 0.001). The Hotelling T^2^ test revealed significant differences between the two groups; the amplitude was significantly lower in the NS compared to the DS nurses (*p* < 0.001, *d* = −1.4, large), while no significant difference in MESOR and acrophase was found between the two groups, though MESOR was slightly lower in the NS nurses.

### 3.2. Rhythmometric Analysis in Night Shift and Day Shift Nurses during the Working Period

**Figure 4:**
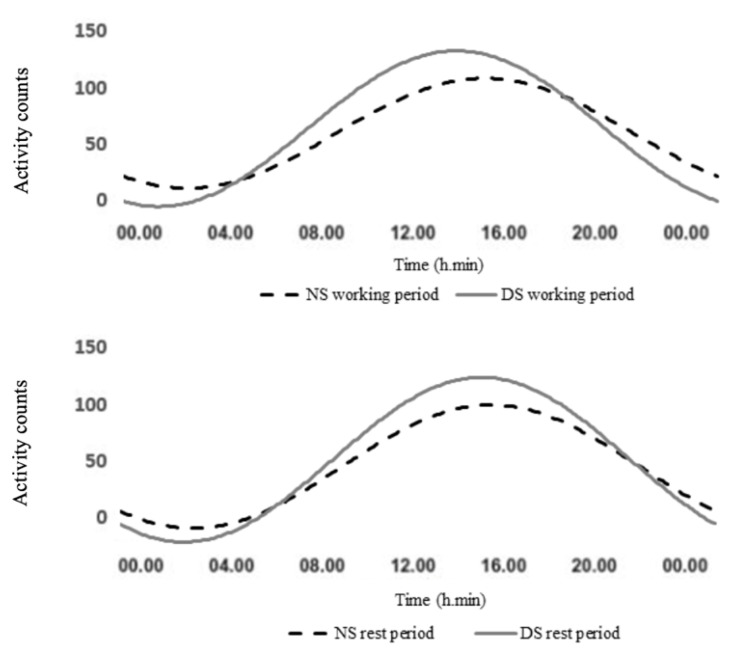
Rest-activity circadian rhythms: data collected by actigraphy in night shift (NS) and day shift nurses (DS) during the working period and the rest period. The dashed black line denotes night shift nurses (NS) for the working and the rest period; the continued grey line denotes day shift nurses (DS) for the working and the rest period.

The population mean cosinor (Table 1) showed a significant circadian rhythm in both groups (*p* < 0.001). The Hotelling T^2^ test revealed significant differences between the two groups during the working period; the amplitude was significantly lower in the NS compared to the DS nurses (*p* < 0.001, *d* = −1.4, large), and the acrophase was significantly different between the two groups (*p* < 0.01, *d* = 1.2, large), with approximately 1 h of delay in the NS group compared to their DS counterparts (Figure 4). No significant difference in MESOR was found between the two groups, though MESOR was slightly lower in the NS nurses.

### 3.3. Rhythmometric Analysis in Night Shift and Day Shift Nurses during the Rest Period

The population mean cosinor (Table 1) showed a significant circadian rhythm in both groups (*p* < 0.001). No significant differences were found by The Hotelling T^2^ test in any of the rhythmometric parameters between the NS and DS nurses during the rest period, though MESOR and amplitude were lower in the NS nurses (Figure 4).

### 3.4. Rhythmometric Analysis between the Working Period and the Rest Period in Night Shift and Day Shift Nurses 

Table 1 shows the comparison between the working period and the rest period in NS and DS nurses. The Hotelling T^2^ test revealed that MESOR and amplitude were significantly higher in the working period compared to the rest period in NS nurses (*p* < 0.001 for both the parameters, MESOR *d* = 1.2, large, amplitude *d* = 0.4, small). Similarly to the DS group, MESOR and amplitude were significantly higher in the working period compared to the rest period (*p* < 0.001, *d* = 1.4, large and *p* < 0.01, *d* = 0.9, large, MESOR and amplitude respectively). 

### 3.5. Rhythmometric Analysis in Normal Weight and Overweight Nurses in Relation to Work Shift during the Entire Working Cycle

Table 2 reports the results of rhythmometric analysis of the normal weight group (<25 kg/m^2^) and the overweight group (≥25 kg/m^2^) during the entire working cycle. The single cosinor method revealed a statistically significant RAR (*p* < 0.001) in all participants.

The population mean cosinor applied to the normal weight and the overweight NS and DS nurses revealed a significant circadian rhythm for both groups (*p* < 0.001). Comparison between normal weight NS and DS nurses showed a significantly lower amplitude for the NS nurses (*p* < 0.001, *d* = −1.2, large). Similarly, in the overweight group, the amplitude was significantly lower for the NS nurses (*p* < 0.001, *d* = −1.3, large). Acrophase and MESOR differed in groups: MESOR was slightly lower in the NS nurses. Table 2 also presents the data for the normal weight and the overweight NS and DS nurses. There were no statistically significant differences in MESOR, amplitude, and acrophase between the two groups. However, MESOR and amplitude were slightly higher for the normal weight NS nurses compared to the overweight NS nurses.

### 3.6. Rhythmometric Analysis in Normal Weight and Overweight Nurses in Relation to Work Shift during the Working Period

The population mean cosinor (Table 2) showed a significant circadian rhythm in both groups (*p* < 0.001). By the Hotelling T^2^ test, significant differences were found between normal weight NS and DS nurses during the working period, showing a significantly lower amplitude for the NS nurses (*p* < 0.01, *d* = −1.2, large). Similarly in the overweight group, the amplitude was significantly lower for the NS nurses during the working period compared to the overweight DS nurses (*p* < 0.001, *d* = −1.2, large), and the acrophase was significantly delayed in NS nurses compared to their DS counterparts in the working period (*p* < 0.001, *d* = 1.7, large).

### 3.7. Rhythmometric Analysis in Normal Weight and Overweight Nurses in Relation to Work Shift during the Rest Period

The population mean cosinor (Table 2) showed a significant circadian rhythm in both groups (*p* < 0.001). No significant difference was found with the Hotelling T^2^ test in any of the rhythmometric parameters between normal weight and the overweight NS and DS nurses during the rest period. 

### 3.8. Rhythmometric Analysis between the Working Period and the Rest Period in Normal Weight and Overweight Nurses in Relation to Work Shift

Table 2 also presents the comparison between the working period and the rest period in normal weight and overweight nurses in relation to work shift. The Hotelling T^2^ test revealed that MESOR was significantly higher in the working period compared to the rest period; it was evident in normal weight NS nurses (*p* < 0.001, *d* = 1.4, large) and in overweight NS nurses (*p* < 0.001, *d* = 1.1, large). Amplitude was significantly higher in the working period compared to the rest period in overweight NS nurses (*p* < 0.05, *d* = 0.4, small).

Similarly in the DS group, MESOR was significantly higher in the working period compared to the rest period: it was evident in normal weight DS nurses (*p* < 0.01, *d* = 1.2, large) and in overweight DS nurses (*p* < 0.01, *d* = 1.6, large). The acrophase was significantly delayed in overweight DS nurses in the rest period compared to their DS counterparts in the working period (*p* < 0.01, *d* = 1.4, large).

## 4. Discussion

For the current study, we evaluated, in a sample of orthopaedic nurses, the effects of working irregular or unusual hours on the rest-activity circadian rhythm. This study suggests alterations in activity levels as monitored by actigraphy in NS and DS nurses. One of the main findings is the difference in RAR between the NS and the DS nurses, considering both the working and the rest period. During the working period, significantly lower amplitude and a delayed acrophase, of approximately 1 h, were observed for the NS compared to the DS counterparts. MESOR was similar for both groups, even if it was slightly lower in the NS compared to the DS nurses.

Since the two groups perform the same type of activities during the working period, the lower activity levels recorded for the NS nurses could be related to their leisure time activity. In fact, during the rest period, the rhythmometric parameters showed no significant differences between the two groups, while MESOR and amplitude were significantly lower compared to the working period in both groups.

Evidence suggests that shift workers are physically less active because of limited time to participate in organized sports activities [45]. In addition, night workers are reported to have more difficulty staying physically fit than other workers [46]. An important factor in the maintenance of physical activity is the psycho-physiological response to exercise. While a shift worker may have the opportunity to participate in some activities, the increased fatigue and negative training experiences caused by misalignment with their biological clock could be reasons why they discontinue recreational sports activity. Another question is whether exercise exacerbates or improves the difficulties that shift workers experience [47]. Workers who generally cope well with shift work may be more inclined to exercise than those who do not. There is evidence that regular physical activity can act as a “synchronizer” by advancing or delaying an individual’s circadian rhythms. Exercise performed at a convenient time of day may help to reduce fatigue, tiredness, sleepiness, and the other short-term effects of shift work [48]. Consistent with these observations, we speculate that the reduction of daytime activity during the rest period in both shift work typologies could be deleterious to health and increase the risk of diabetes and obesity [49], cancer [50], neurodegenerative diseases [21], and cardiovascular diseases [22,23].

Referring to the acrophase, despite the data showed a delay of approximately 1 h in NS compared to DS nurses during the working period, it does not seem that the circadian rhythm regularity was affected by the night shift work. Probably a single night planned on a 5-day shift schedule is not able to change the worker’s circadian rhythm.

Moreover, shift work can also interfere with metabolism. Our data showed that, independent of BMI, the amplitude was significantly lower in the NS compared to the DS nurses during the working period. BMI may influence RAR in night shift workers, who are noted to have a higher risk of illness, but this relationship was not completely clear due to the small sample size. Previous studies found a correlation between BMI and circadian disruption [51,52,53,54,55,56]; it seems that this relationship may be considered bidirectional. Obesity can increase the risk of abnormalities in RAR, and circadian disruption can interfere with the metabolic and nutritional balance of the body. Eating at an inappropriate time in the circadian rhythm (i.e., at night in humans), as shift workers commonly do, may increase the risk of obesity due to a misalignment between central and peripheral clocks [54]. In addition, changes in normal eating behaviors (e.g., eating energy-dense snacks) can also interfere with maintaining a correct energy balance [57]. Furthermore, disruption of circadian rhythm could result from the suppression of melatonin secretion caused by exposure to night light, which is one of the key factors in the development of obesity in night shift workers. Evidence suggests that melatonin plays a central role in synchronizing central and peripheral circadian rhythms and regulates the secretion of hormones (e.g., cortisol, insulin, leptin). Such misalignment may lead to a disturbance in body homeostasis and result in an abnormal metabolic profile [51,56].

The limitations of the present study include the small sample size. Additional information (e.g., marital status, number of children or care of elderly family member) could have provided greater detail about the sample and the activity levels associated with daily housework and family commitments. In addition, data collection around dietary habits or stress measurements could have supplied a better interpretation of how night shift work might affect nursing staff more generally.

## 5. Conclusions

Based on the data, the strength of the present study is the objective measurement of RAR by actigraphy in the comparison of nurses working night and day shifts. The findings underscore the importance of further investigations evaluating the relationship between activity levels and shift work.

In this perspective, future studies are needed to determine whether physical activity programs, scheduled in the nurses’ leisure time or conducted at the workplace, could improve circadian activity levels and prevent alterations in the sleep-wake cycle in shift workers.

## Figures and Tables

**Table 1:** Population mean cosinor analysis in night shift and day shift nurses during the entire working cycle, the working period and the rest period.

Group	PR (%)	*p*-Value	MESOR (a.c.)	Amplitude (a.c.)	Acrophase (h:min)
NS (*n* = 44)					
Entire working cycle	28	<0.001	113.1 ± 7.57	59.9 ± 6.2 ^a^	15:34 ± 00:48
Working period	10	<0.001	122.7 ± 8.5 ^d^	67.5 ± 6.9 ^b,e^	14:32 ± 00:31 ^c^
Rest period	11	<0.001	87.9 ± 8.8 ^d^	51.5 ± 10.5 ^e^	14:52 ± 01:11
DS (*n* = 15)					
Entire working cycle	47	<0.001	120.9 ± 14.53	90.6 ± 14.26 ^a^	15:09 ± 01:25
Working period	21	<0.001	128.5 ± 15.5 ^f^	96 ± 14.5 ^b,g^	13:29 ± 02:31 ^c^
Rest period	14	<0.001	90.3 ± 17.1 ^f^	69.8 ± 20.8 ^g^	14:40 ± 01:22

Analysis of RAR in night shift and day shift nurses during the entire working cycle, the working period and the rest period. Data are expressed as mean ± 95% CI. NS: night shift nurses. DS: day shift nurses. PR: percentage of rhythm. MESOR (activity counts): Midline Estimating Statistic of Rhythm. Amplitude (activity counts): half the difference between the highest and the lowest points of the cosine function best fitting the data. Acrophase (h:min): time in which the highest value falls. Significances of Hotelling T^2^ test: ^a^ *p* < 0.001: difference in amplitude during the entire working cycle between NS and DS group. ^b^ *p* < 0.001: difference in amplitude during the working period between NS and DS group. ^c^ *p* < 0.01: difference in acrophase during the working period between NS and DS group. ^d^ *p* < 0.001: difference in MESOR in NS group between the working period and the rest period. ^e^ *p* < 0.001: difference in amplitude in NS group between the working period and the rest period. ^f^ *p* < 0.001: difference in MESOR in DS group between the working period and the rest period. ^g^ *p* < 0.01: difference in amplitude in DS group between the working period and the rest period.

**Table 2:** Population mean cosinor analysis in normal weight and overweight nurses in relation to work shift during the entire working cycle, the working period and the rest period.

Group	PR (%)	*p*-Value	MESOR (a.c.)	Amplitude (a.c.)	Acrophase (h:min)
BMI < 25 kg/m^2^					
Entire working cycle					
NS (*n* = 19)	29	<0.001	117.3 ± 12.6	62 ± 11 ^a^	15:47 ± 01:21
DS (*n* = 9)	51	<0.001	119.8 ± 14.68	92 ± 16.89 ^a^	15:31 ± 02:07
Working period					
NS (*n* = 19)	11	<0.001	128.3 ± 13 ^f^	71 ± 10.7 ^c^	14:39 ± 01:04
DS (*n* = 9)	21	<0.001	126.3 ± 21.4 ^i^	99 ± 24.7 ^c^	13:35 ± 02:32
Rest period					
NS (*n* = 19)	11	<0.001	88.1 ± 13.3 ^f^	52.9 ± 19.6	15:08 ± 01:03
DS (*n* = 9)	17	<0.001	101.7 ± 18.3 ^i^	81 ± 26.8	14:49 ± 02:01
BMI ≥ 25 kg/m^2^					
Entire working cycle					
NS (*n* = 25)	27	<0.001	109.8 ± 9.89	58.4 ± 7.66 ^b^	15:47 ± 01:02
DS (*n* = 6)	41	<0.001	122.5 ± 38.39	90 ± 33.83 ^b^	15:39 ± 02:14
Working period					
NS (*n* = 25)	10	<0.001	118 ± 11.7 ^g^	64.7 ± 9.7 ^d,h^	14:25 ± 01:38 ^e^
DS (*n* = 6)	18	<0.001	131.4 ± 37.1 ^l^	97.7 ± 24.7 ^d^	12:26 ± 02:07 ^e,m^
Rest period					
NS (*n* = 25)	10	<0.001	87.8 ± 12.6 ^g^	49.8 ± 12.1 ^h^	14:38 ± 01:59
DS (*n* = 6)	11	<0.001	75.2 ± 35.2 ^l^	55 ± 41.3	14:21 ± 04:59 ^m^

Actigraphy-based analysis of RAR in normal weight (BMI < 25 kg/m^2^) and overweight nurses (BMI ≥ 25 kg/m^2^) in relation to work shift during the entire working cycle, the working period, and the rest period. Data are expressed as mean ± 95% CI. NS: night shift nurses. DS: day shift nurses. PR: percentage of rhythm. MESOR (activity counts): midline estimating statistic of rhythm. Amplitude (activity counts): half the difference between the highest and the lowest points of the cosine function best fitting the data. Acrophase (h:min): time in which the highest value falls. Significances of Hotelling T^2^ test: ^a^ *p* < 0.001: difference in amplitude during the entire working cycle between normal weight NS and DS group. ^b^ *p* < 0.001: difference in amplitude during the entire working cycle between overweight NS and DS group. ^c^ *p* < 0.01: difference in amplitude during the working period between normal weight NS and DS group. ^d^ *p* < 0.001: difference in amplitude during the working period between overweight NS and DS group. ^e^ *p* < 0.001: difference in acrophase during the working period between overweight NS and DS group. ^f^ *p* < 0.001: difference in MESOR between the working and the rest period in normal weight NS group. ^g^ *p* < 0.001: difference in MESOR between the working and the rest period in overweight NS group. ^h^ *p* < 0.05: difference in amplitude between the working and the rest period in overweight NS group. ^i^ *p* < 0.01: difference in MESOR between the working and the rest period in normal weight DS group. ^l^ *p* < 0.01: difference in MESOR between the working and the rest period in overweight DS group. ^m^ *p* < 0.01: difference in acrophase between the working and the rest period in overweight DS group.

## Data Availability

All data generated or analyzed during this study are included in this article.
